# Male Sterility is linked to the Flavonoid Biosynthesis Pathways in Prunus mira

**DOI:** 10.6026/97320630016363

**Published:** 2020-05-31

**Authors:** Shanshan Zhang, Gesang Pingcuo, Hong Ying, Fan Zhao, Yongning Cui, Xiuli Zeng

**Affiliations:** 1 The ministry of agriculture of Qinghai-Tibet plateau fruit trees scientific observation test station, Lhasa Tibet, 850032, China; 2 Institute of Vegetables, Tibet Academy of Agricultural and Animal Husbandry Sciences, Lhasa, Tibet, 850002, China

**Keywords:** Plant floral fertility, metabolome, domestication, gene expression profiling

## Abstract

Sterility plays an important role in plant adaptation and evolution and has contributed to the development of high yielding crop hybrids. We used the widely targeted metabolomics profiling to survey the metabolites and biological pathways associated with male sterility in Prunus mira by comparing flowers from fertile and sterile trees. Male sterile flowers displayed abnormal stamen, uncolored anthers, and distorted and shrunken pollen grains with an apparent lack of turgidity. We report 566 metabolites in six flower samples and 140 differentially accumulated metabolites (DAMs) between both flower types. Most of the DAMs belong to the phenyl propanoid biosynthesis pathway, particularly flavonoid, flavone and flavonol biosynthesis pathways, implying that alterations in these key pathways link to male sterility in P. mira. The known link between low levels of flavonoid metabolites, weak expression levels of several structural genes from the phenyl propanoid biosynthesis pathway and hyper accumulation of reactive oxygen species were highlighted for understanding the underlying mechanism leading to the abnormal or aborted pollen grains observed in the sterile flowers. Data on the molecular mechanism of male sterility in Prunus mira will facilitate further in-depth investigations on this important agronomic and ecological trait.

## Background

Prunus mira Koehne syn. Amygdalus mira is an important wild fruit tree in China endowed with special economic, medicinal and ecological properties. It is used for vegetation restoration and soil erosion control in the Tibet plateau [1]. Prunus mira fruit is employed in Chinese traditional medicine as remedy for irregular menstruations, fracture and congestion [2]. Moreover, the fruit is a reservoir of bioactive nutrients such as vitamins A, B, C, fibers and potassium, phenolic acids and flavonoids. [3]. Besides, P. mira has the most ornamental value among major wild Prunus species [4]. Owing to its long-term adaptation to the high stressful conditions in the Tibet plateau, P. mira has developed strong resistance to several biotic and abiotic stresses [5]. Efforts are underway for the domestication of the species, particularly in the Tibet region and for its use as an important genetic resource for the improvement of cultivated peach [4,6]. In a previous field survey,Zeng [7] observed that many wild P. mira trees in the Tibet region were male sterile. The high proportion of male sterility trait may have contributed to the high genetic variation observed in P. mira [8,9].

The loss of the male function plays an important role in plant adaptation and evolution. Darwin [10] suggested that this natural phenomenon enhances adaptation through gene transfer from various related and unrelated individuals. On the other hand, male sterility has immensely contributed to the development of high yielding crop hybrids. Heterosis or hybrid vigor is a widespread phenomenon in plant species [11] and refers to the ability of the F1 hybrid to outperform either of the parents used in the cross. Hybrid seed production is one the most valuable techniques to meet the global food demand [12]. Heterosis systems have been used to increase yields in several crops species such as rice, soybeans, barley, cotton, eggplant, maize and rapeseed [13-19].

Metabolomics is a powerful technique because metabolites and their concentrations directly reflect the underlying biochemical activity and metabolic state of cells, tissues, or organisms [20]. In recent years, it has been extensively applied to clarify gene functions and elucidate the molecular basis of important agronomic and economic traits in plants [21-25]. Several studies have documented that disruptions in plant secondary metabolites such as flavonoids, lipids, glutathione are associated with male sterility trait in plants [26-28], therefore metabolomics represents a promising tool to study male sterility trait in Prunus mira.

It is of interest to elucidate the molecular basis of male sterility trait in Prunus mira given its economic and ecological importance. Hence, we profiled the floral metabolome of male sterile and fertile P. mira trees. Comparative analysis of the metabolite concentration showed the key metabolites and pathways involved in male sterility trait in P. mira. We believe that these data have applications in the domestication, cultivation and breeding of P. mira.

## Materials and Methods

### Plant materials and flower sampling

**Figure 1:**
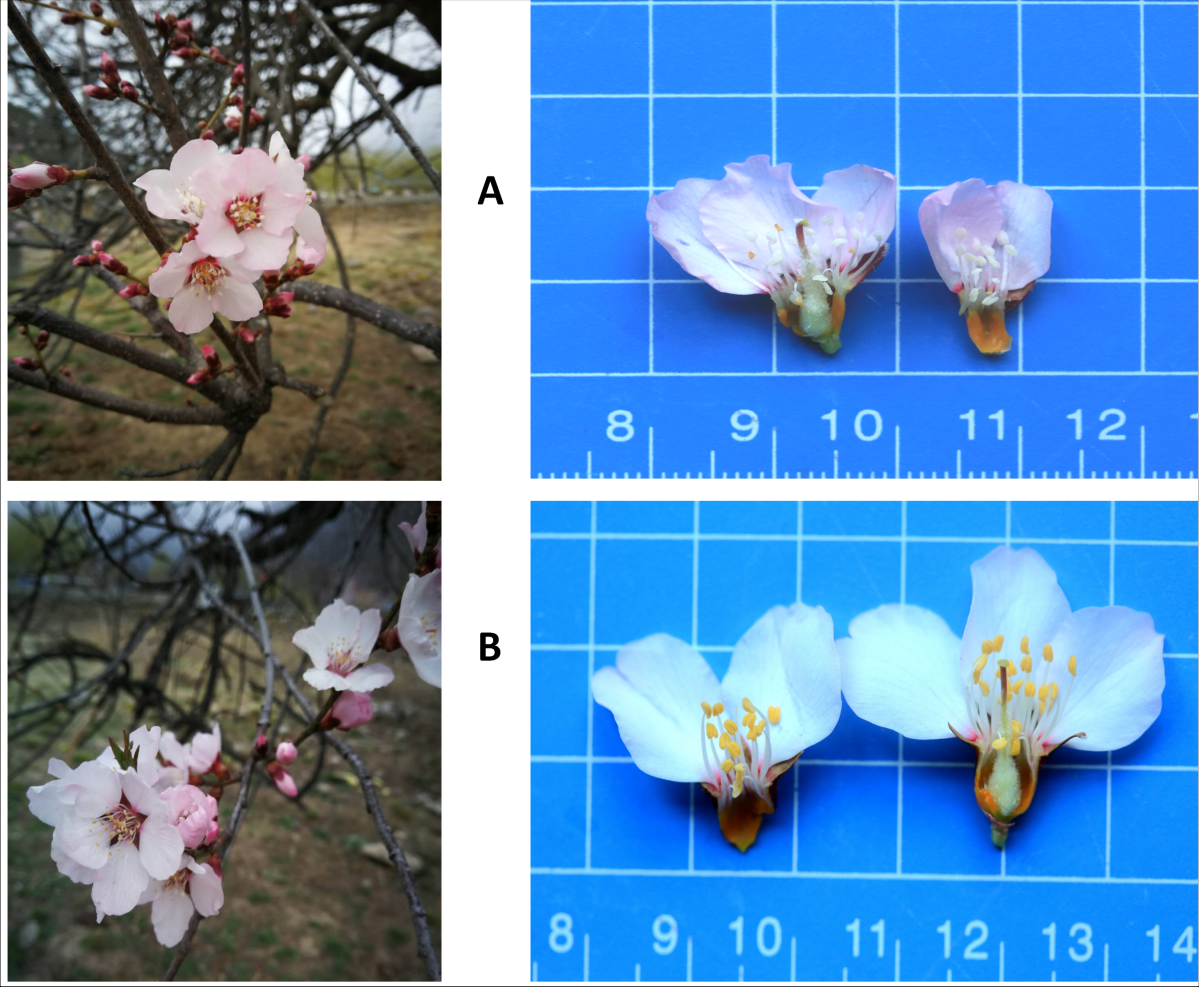
Photos of the Prunus mira flowers. (A) Male sterile flowers (SF) showing abnormal stamen development and uncolored anthers; (B) Fertile flowers (FF) displaying normally developed stamen and colored anthers.

The study was conducted in the Bomê County, Nyingchi City, Tibet Autonomous Region, China (29.850°N latitude, 95.775°E longitude). Samples were collected in March-April 2018, which is the Prunus mira Koehne blossom season. Flower collections were made on adult trees with more than 100 years old, including a male sterile tree and a male fertile tree. The male sterile flowers could be visually distinguished from the male fertile flowers by their abnormal stamen and uncolored anthers (Figure 1). The flower samples were composed of the stamens and pistils. On each tree, we randomly picked 45 flowers from different parts of the tree and constituted three biological replicates. In total, six samples were collected, frozen immediately in liquid nitrogen in the field, transported to the laboratory and then stored at -80°C until further use.

### Scanning electron microscopy (SEM)

For SEM analysis, anthers and pollen grain samples were gold coated on a Sputter coater (Emitech, Houston, USA). Photographs were taken on the scanning electron microscope Phillips ESEM; model XL30 at an intensity of 15 KV [36].

### Metabolic profiling

The sample preparation, extract analysis, metabolite identification and quantification were performed at Wuhan MetWare Biotechnology Co., Ltd. (www.metware.cn) following their standard procedures and previously fully described by Yuan et al. [29] and Zhang et al. [58].

### Metabolite data analysis

Before the data analysis, quality control (QC) analysis was conducted to confirm the reliability of the data. The QC sample was prepared by the mixture of sample extracts and inserted into every two samples to monitor the changes in repeated analyses. Data matrices with the intensity of the metabolite features from the six samples were uploaded to the Analyst 1.6.1 software (AB SCIEX, Ontario, Canada) for statistical analyses. The supervised multivariate method, partial least squares-discriminant analysis (PLS-DA), was used to maximize the metabolome differences between the pair of samples. The relative importance of each metabolite to the PLS-DA model was checked using the parameter called variable importance in projection (VIP). Metabolites with VIP ≥ 1 and fold change ≥ 2 or fold change ≤ 0.5 were considered as differential metabolites for group discrimination [29]. Principal component analysis and hierarchical clustering heatmap were performed in the R software (www.r-project.org).

### Quantitative Reverse Transcriptase PCR (qRT-PCR)

The SYBR Premix Ex Taq™ Kit (Takara, Dalian, China) was employed for the qRT-PCR following to the manufacturer's instructions. The qRT-PCR was conducted on the StepOne plus Real time PCR Platform (Applied Biosystems, CA, USA) with the following protocol: 95°C for 10 min, followed by 40 cycles of 95°C for 15 s, and at 60°C for 60 s [59]. Each reaction was performed using a 20 µL mixture containing 10 µL of 2 X ChamQ SYBR qPCR Master Mix, 6 µL of nuclease-free water, 1 µL of each primer (10 mM), and 2 µL of 4-fold diluted cDNA. Specific primer pairs of phenylpropanoid biosynthesis structural genes were designed (Table S3). The Actin gene was used as the internal control. Data are presented as relative transcript levels based on the 2^−ΔΔCt^ method [60].

### Measurement of malondialdehyde content

Malondialdehyde (MDA) content in the anther samples was measured using Nanjing Jiancheng Bioengineering Institute's relevant kit following manufacturer's instructions. Data were analyzed with the R software (www.r-project.org) using the one-way analysis of variance (ANOVA) for significant difference. The error bars were calculated with data from three replicates. ANOVA results were considered significant at P < 0.05 and mean comparisons were done using the Tukey HSD test.

## Results 

### Morphological characteristics of Prunus mira anthers and pollen grains

**Figure 2:**
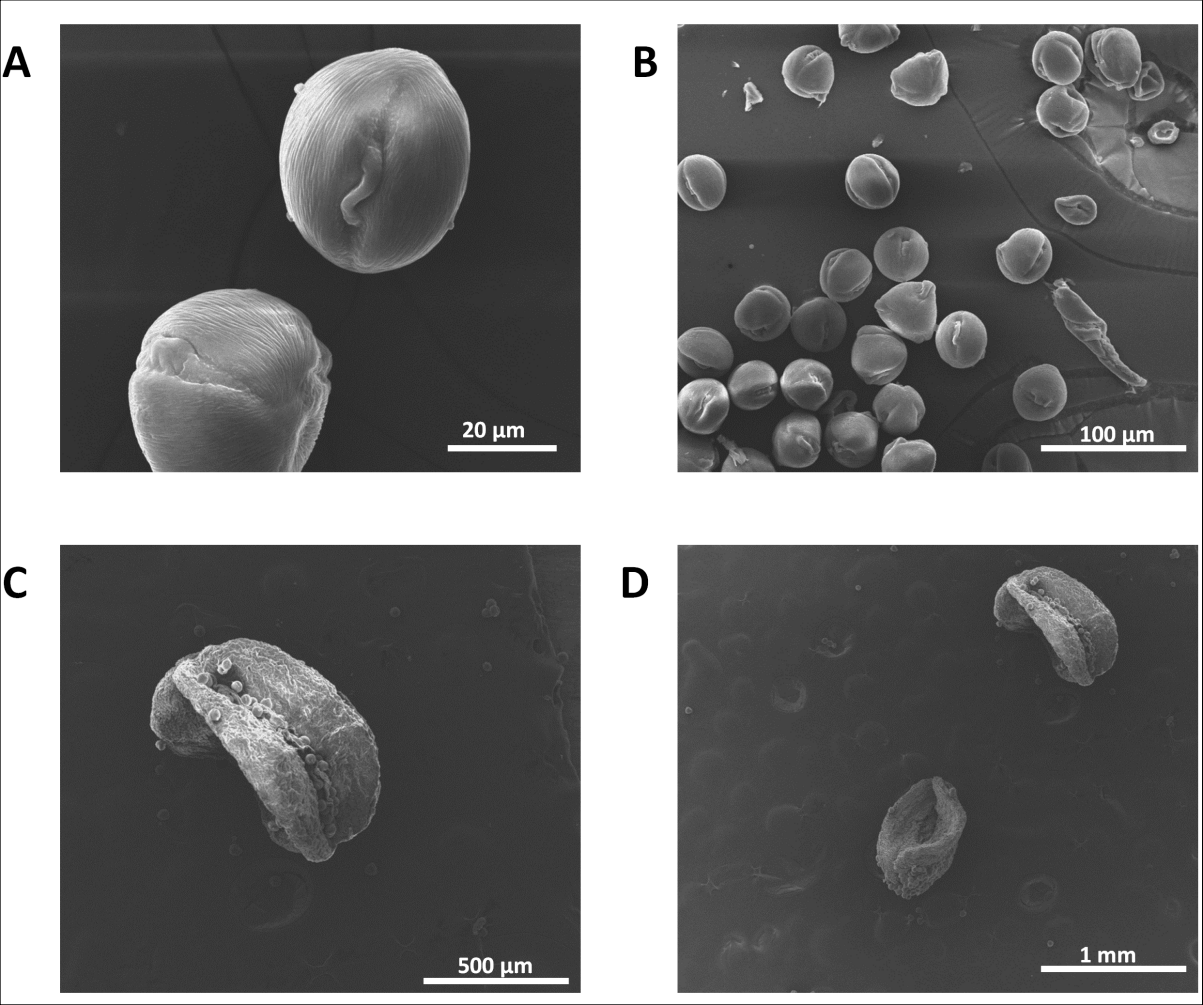
SEM pictures of pollen grains in anthers of fertile and sterile flowers at different magnifications. (A,B) fertile pollen; (C,D) sterile pollen.

In the present study, we analyzed the metabolite profiles in flower samples from two types of Prunus mira trees: male fertile and male sterile. The male sterile flowers could be visually distinguished from the male fertile flowers by their abnormal stamen and uncolored anthers (Figure 1). We named as FF and SF the flower samples from the male fertile and male sterile trees, respectively. We performed scanning electron microscopy (SEM) analyses of the anther and pollen grain samples from both flower types. As shown in (Figure 2), the wall morphology and the abundance of pollen grains from the two flower types were different. The outer surface of pollen grains from the fertile anthers displays globoid shape, smooth and regular perforated orientation over the exine while the pollen of the sterile plants showed distorted and shrunken morphology and an apparent lack of turgidity (Figure 2A and 2C). In addition, the pollen grains were abundant in the anthers of the fertile flowers, whereas very few pollen grains could be observed in the anthers of the sterile flowers (Figure 2B and 2D).

### Overview of the metabolite profiles in Prunus mira flowers

**Figure 3:**
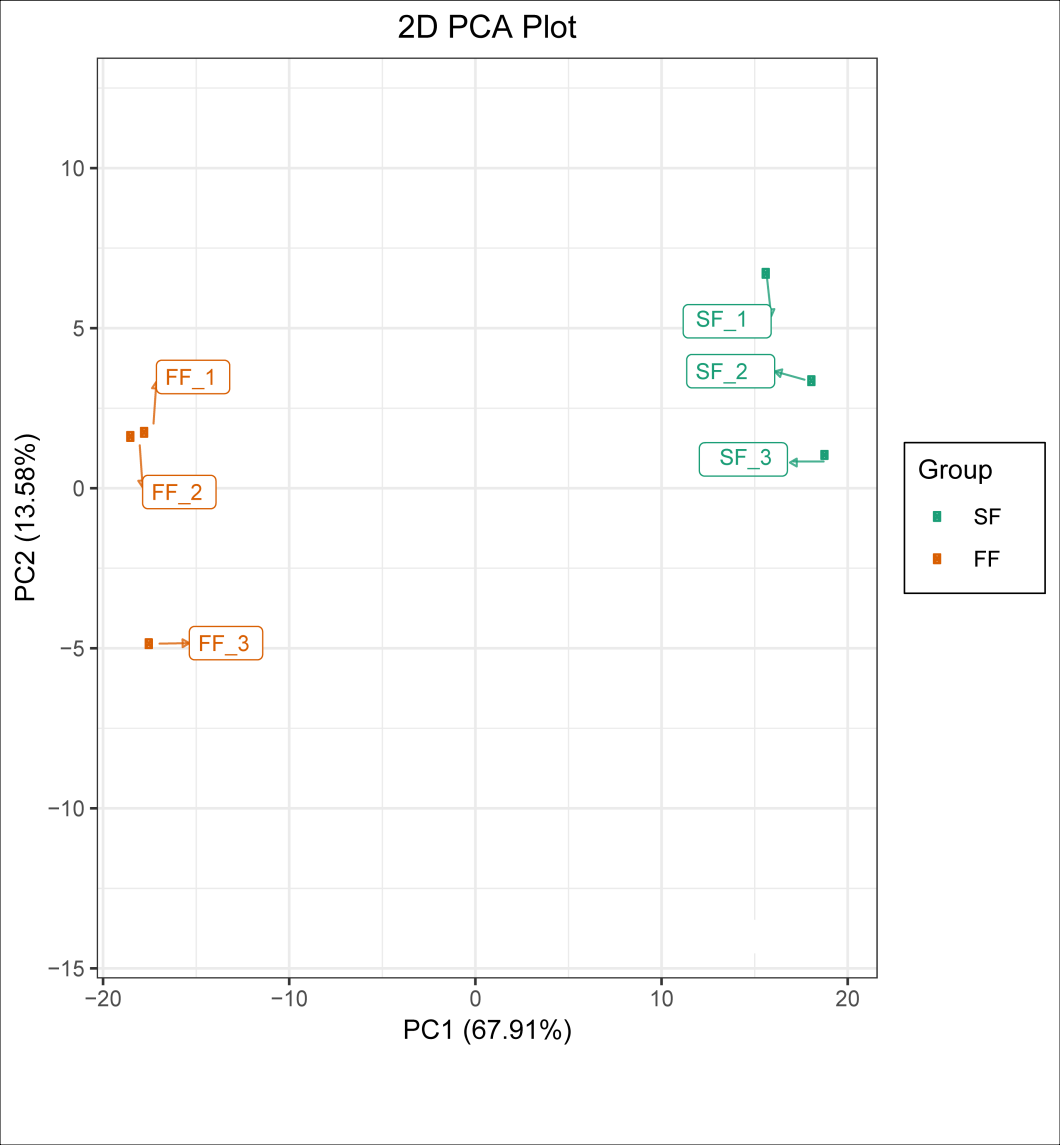
Overview of global metabolic profiles in flower samples of Prunus mira. Principal component analysis. Samples represent fertile flowers (FF), and sterile flowers (SF). Data represent the log2 fold change of the metabolite ion intensity.

A total of six flower samples were used for metabolite detection and quantification by employing the widely-targeted metabolomics approach. We identified 566 metabolites in the flowers samples. The metabolites detected were classified into 32 major classes, predominantly organic acids, amino acid derivatives and nucleotide and its derivatives (Table 1; Table S1). Based on the quantification of the different metabolites principal component analysis (PCA) was performed. As shown in (Figure 3), all the biological replicates were clustered together highlighting a good correlation between replicates and the high reliability of our data. SF samples were clearly distinguished from FF samples and the first two PCs could explain over 80% of the total variation.

### Differentially accumulated metabolites between flowers from male sterile and male fertile trees

**Figure 4:**
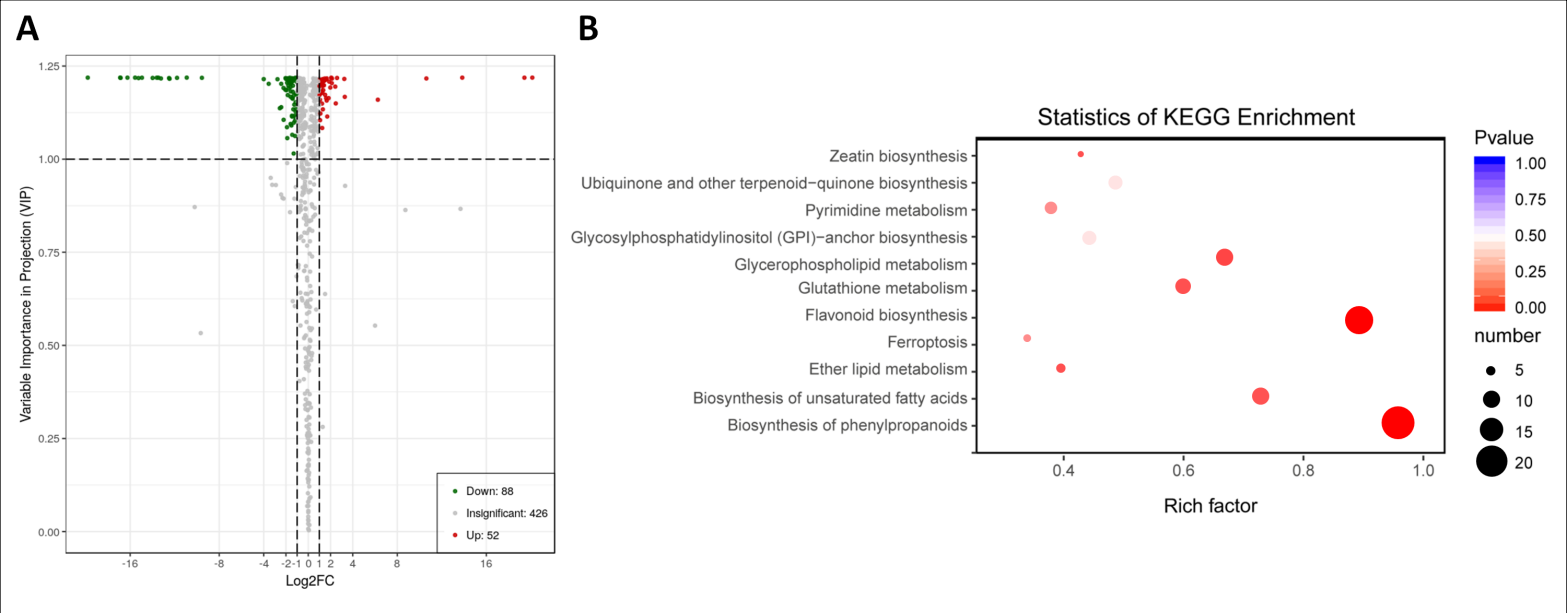
Differentially accumulated metabolites (DAM) between male sterile (SF) and fertile (FF) flowers. (A) Volcano plot showing the up and down-regulated genes between SF and FF; (B) KEGG enrichment analysis of the DAMs identified between SF and FF

We compared the metabolite ion intensity in flowers of SF to FF, in order to determine the differentially accumulated metabolites (DAM) based on the variable importance in projection (VIP) ≥ 1 and fold change ≥ 2 or fold change ≤ 0.5 [29]. We identified 140 DAMs including 88 down- and 52 up-accumulated metabolites in the male sterile flowers (Figure 4A;Table S2). The DAMs belong to 25 different classes, implying that male sterility trait is caused by alterations in various biological pathways. Nonetheless, kyoto encyclopedia of genes and genome (KEGG) enrichment analysis showed that these DAMs were mainly enriched in the phenyl propanoid biosynthesis pathway, in particular flavonoid, flavone and flavonol biosynthesis related pathways (Figure 4B). This result highlights the primordial role of flavonoids in pollen formation and fertility in Prunus mira.

### Differentially accumulated metabolites mapped to the phenyl propanoid-flavonoid pathways

**Figure 5:**
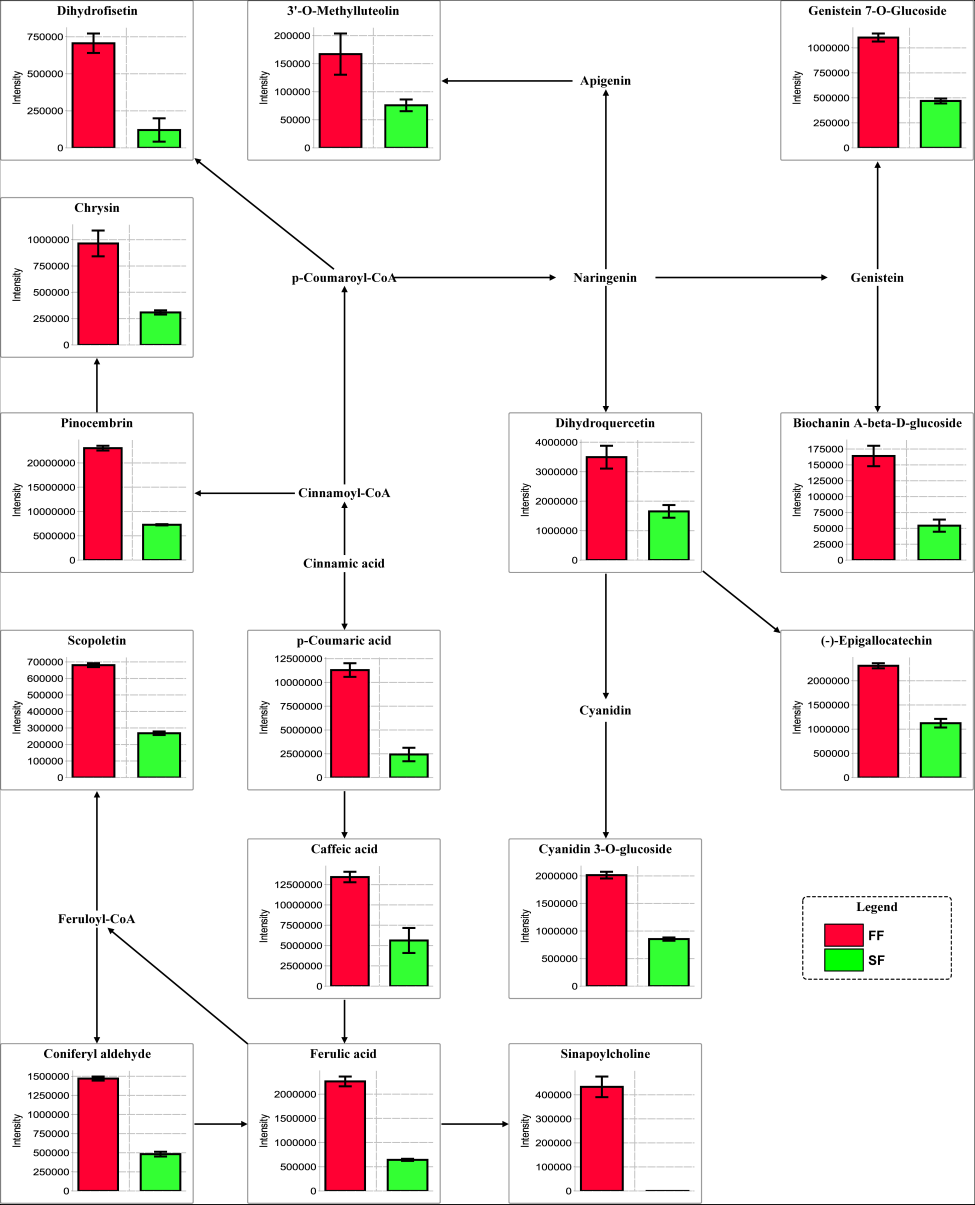
Metabolic pathways of the differentially accumulated metabolites between sterile flowers (SF) and fertile flowers (FF) mapped to the phenylpropanoid-flavonoid pathways in Prunus mira. Metabolite ion intensity was displayed.

In order to clarify how changes in the concentration of the phenyl propanoid-flavonoid related metabolites impact on flower fertility, we searched and mapped all the annotated DAMs involved in the phenyl propanoid-flavonoid pathways. Several compounds could not be placed on the phenyl propanoid-flavonoid pathway because of lack of annotation but the 15 compounds that were successfully mapped clearly showed a higher accumulation in fertile flower samples as compared to sterile flowers (Figure 5). These results suggest that high accumulation of flavonoid compounds may be a key mechanism for floral fertility in Prunus mira.

### Expression profiling of the genes involved in the phenylpropanoid-flavonoid pathways

**Figure 6:**
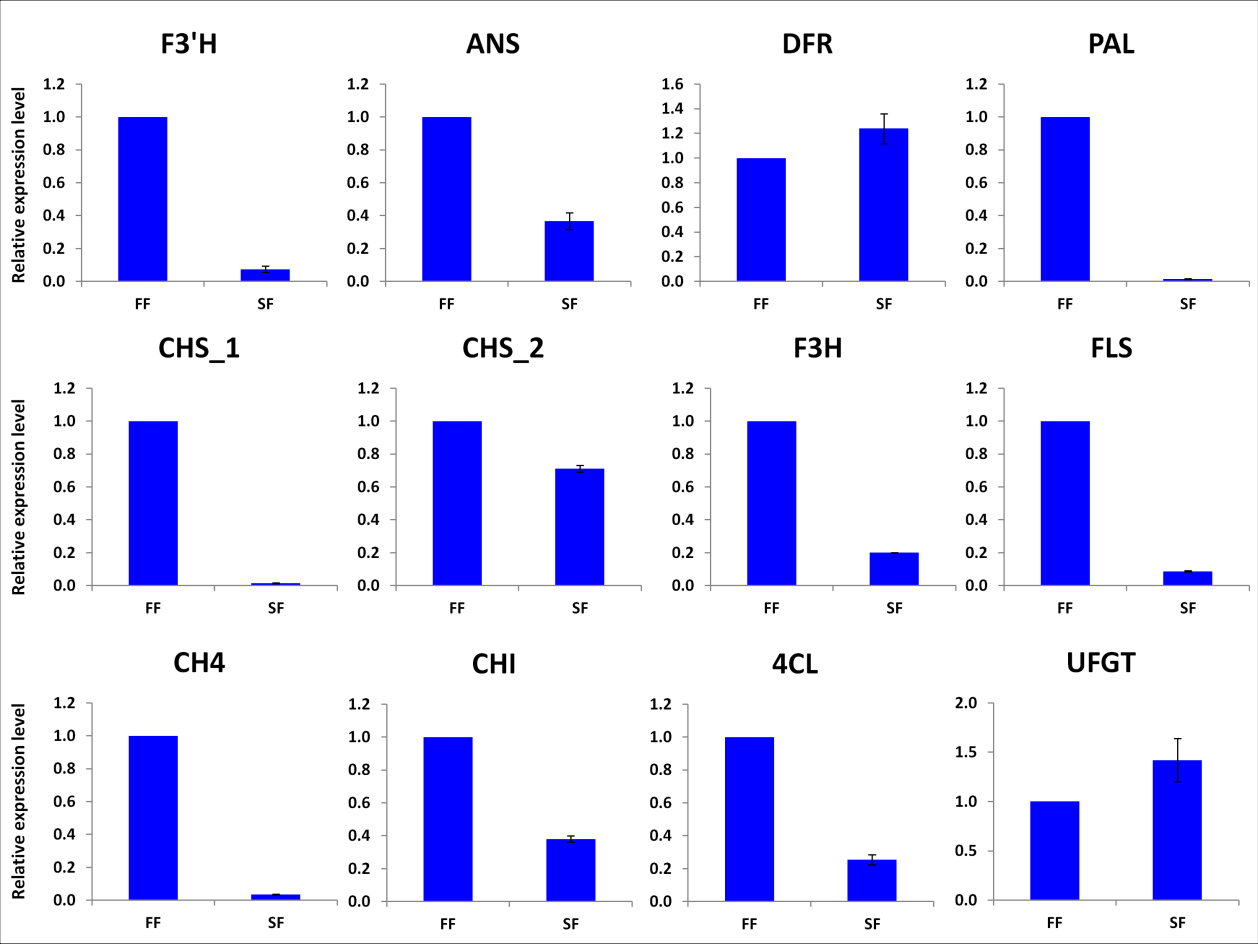
qRT-PCR expression profiling of 12 structural genes involved in the phenylpropanoid-flavonoid pathways. Phenylalanine ammonia-lyase (PAL), chalcone synthase (CHS), chalcone isomerase (CHI), flavonone 3-hydroxylase (F3H), flavonoid 3'-monooxygenase (F3'H), dihydroflavonol 4-reductase (DFR), anthocyanin synthase (ANS), and UDP-glucose-flavonoid 3-O-glucosyltrasnferase (UFGT),cinnamic acid 4-hydroxylase (C4H); 4 coumarate CoA ligase (4CL), flavonol synthase (FLS). FF and SF stand for fertile and sterile flowers,respectively. The error bar represents the SD of three biological replicates. The Actin gene was used as the internal reference gene for normalization.

Various structural genes catalyze the synthesis of diverse metabolites in the phenylpropanoid biosynthesis pathways. We selected 12 genes, including phenylalanine ammonia-lyase (PAL), chalcone synthase (CHS), chalcone isomerase (CHI), flavonone 3-hydroxylase (F3H), flavonoid 3'-monooxygenase (F3'H), dihydroflavonol 4-reductase (DFR), anthocyanin synthase (ANS),and UDP- glucose-flavonoid 3-O-glucosyltrasnferase (UFGT), cinnamic acid 4-hydroxylase (C4H); 4 coumarate CoA ligase (4CL), flavonol synthase (FLS) and compared their transcript levels in SF and FF samples based on quantitative Reverse Transcriptase PCR. Ten genes displayed significant different transcript levels between the two samples (Figure 6). Overall, we observed a down-regulation of the majority of the investigated structural genes, which matched, with the low accumulation of phenylpropanoid-flavonoids compounds in SF (Figure 5).

### Malondialdehyde content in SF and FF anthers

**Figure 7:**
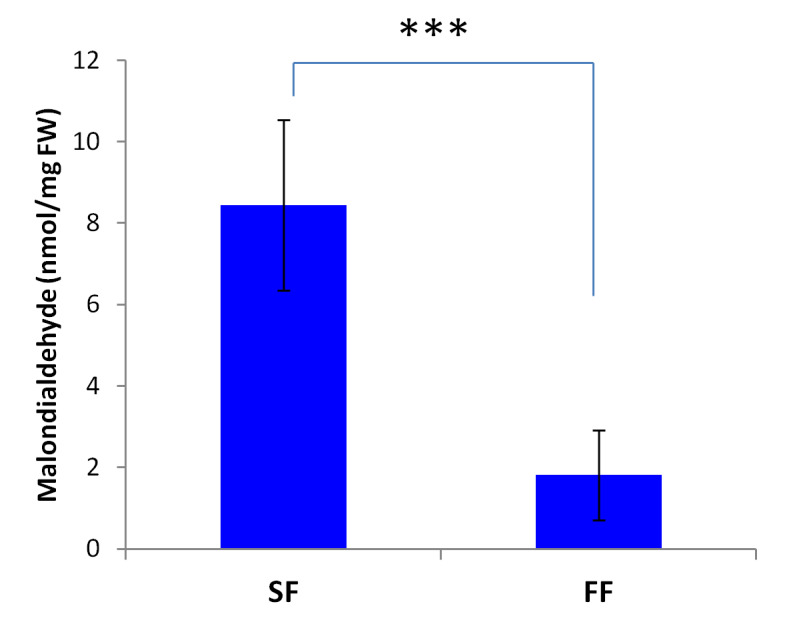
MDA content in Prunus mira fertile flower (FF) and sterile flowers (SF). Error bar represent SD from three replicate values. *** mean values significantly different at P < 0.001.

It has been reported that high accumulation of reactive oxygen species (ROS) in anthers leads to pollen abortion and male sterility in crops [30, 31]. Malondialdehyde (MDA) content is associated with lipid peroxidation via an increased generation of ROS [32]. Hence, high level of MDA is an indicator of high stress damage. By comparing the two flower types, we found that the level of MDA was significantly higher in SF as compared to FF (P < 0.001), indicating high level of ROS in SF anthers (Figure 7).

## Discussion:

Male sterility is an important trait in crop breeding and for the conservation of natural genetic diversity in plants [10,33, [34]. Although male sterility has been reported in some plant species, a natural occurring system is not available or when available is nor often usable for several plant species, including important agricultural crops [35,36]. A previous investigation on flower diversity from various wild Prunus mira trees in the Tibet (China) revealed that many trees were male sterile but are able to produce fruits [7]. Such male sterility system in Prunus mira is potentially exploitable and the goal of this study was to elucidate its molecular basis. With the gradual domestication of Prunus mira, the male sterility trait could be a key technological approach in the development of hybrid seed. In plants, male sterility is caused by various mechanisms related to anther abortion, defect in pollen morphology, number, size, shape, etc. [13]. Our study showed that the sterile flowers had abnormal pollen grains and importantly the formation of pollen grains was significantly impaired, which is similar to observations in tobacco [36].

We observed that many of the potential metabolites associated with male sterility trait were related to the phenyl propanoid biosynthesis pathway, particularly the flavonoid and flavone and flavonol biosynthesis pathways. This implies that alterations in these pathways may cause male sterility in P. mira. More importantly, the differential metabolite accumulation between the two flower samples matched well with the transcript levels of some structural genes. Our results corroborate the conclusions of van der Meer et al. [37] who demonstrated that an antisense inhibition of the chalcone synthase gene from the flavonoid biosynthesis in Petunia anthers results in male sterility. Besides, several studies have shown that flavonols are required for male fertility and more specifically for the pollen tube growth [26-28]. Also, it has been demonstrated that flavonoid biosynthesis pathway is involved in the process of forming the pollen coat in various plant species such as Arabidopsis, Brassicas and cotton [38-41]. Ingrosso et al. [42] over-expressed the stilbene synthase gene in tomato and obtained a complete male sterility due to significant impairment in the flavonoid metabolism. A recent study discovered that most genes encoding key enzymes in the phenylpropanoid biosynthesis pathway were found significantly altered between male sterile and male fertile wheat cultivars [43]. Altogether, our conclusion is in line with these numerous reports showing that metabolic disruptions in the flavonoid pathways affect pollen grain formation and fertility in Prunus mira. Besides, several altered metabolites belonging to other pathways, including lipids, carbohydrates, organic acids, may also play important roles for male sterility in Prunus mira as shown in other plant species [38, 44-46].

It has been reported that abnormal accumulation of reactive oxygen species (ROS) in plant anthers damages lipids, proteins and DNA, inhibits enzyme activity, activates programmed cell death pathway, and causes male sterility [30,31,47]. Flavonoids and other antioxidant molecules play a crucial role in pollen development and protection against ROS [48]. We observed a striking high level of ROS indicator (malondialdehyde) in Prunus mira sterile flowers (SF) compared to the fertile ones, while low levels of flavonoid metabolites and low expression levels of several structural genes were detected in SF. This suggests that reduced flavonoid levels could not efficiently buffer ROS accumulation and induced damages in FS anthers, which may be the cause of the male sterility in Prunus mira, similar to conclusions reported in rice, cotton, pepper, wheat, and soybean [49-54].

The expression levels of structural genes involved in the phenylpropanoid pathways are regulated by transcription factors (TF) such as MYB and bHLH [55, 56]. Ying et al. [57] recently de novo assembled the transcriptome of Prunus mira fruit samples and showed that the structural genes involved in the phenylpropanoid pathways are regulated by hundreds of potential TFs. To clarify the complete genetic network involved in the male sterility trait in Prunus mira, further studies related to transcriptome sequencing in flower samples from various male sterile and fertile trees are needed. Furthermore, integrating such transcriptomic information with our metabolomic data will better facilitate the elucidation of the molecular basis of male sterility in Prunus mira [57].

## Conclusions:

We report the metabolic profiles of flower samples from male sterile and fertile Prunus mira trees. Comparative analysis helps identify potential metabolites and enriched metabolic pathways associated with the male sterility trait in P. mira. Data reported here along with a transcriptome analysis will help link the relationship between the candidate metabolites, genes and biological pathways involved in male sterility trait in Prunus mira. We believe a comprehensive analysis on the P. mira male sterility would help understand conservation of genetic diversity and breeding efforts in both wild and cultivated peach species.

## Figures and Tables

**Table 1:** Classification of the 603 detected metabolites into major classes

Class	Number of compounds	Class	Number of compounds
Organic acids	64	Lipids_Glycerolipids	13
Amino acid derivatives	53	Vitamins	12
Nucleotide and its derivates	51	Catechin derivatives	11
Flavone	38	Isoflavone	10
Lipids_Glycerophospholipids	32	Anthocyanins	8
Flavonol	31	Phenolamides	8
Amino acids	25	Alcohols and polyols	7
Hydroxycinnamoyl derivatives	25	Indole derivatives	6
Others	25	Cholines	5
Quinate and its derivatives	22	Proanthocyanidins	5
Carbohydrates	18	Tryptamine derivatives	5
Flavanone	17	Alkaloids	3
Flavone C-glycosides	17	Nicotinic acid derivatives	3
Lipids_Fatty acids	17	Pyridine derivatives	2
Coumarins	16	Flavonolignan	1
Benzoic acid derivatives	15	Terpenoids	1

## References

[1] Hao HP (2009). J Plant Ecol..

[2] Dong GZ (1991). Quart For by-product Spec China.

[3] Liu L, Meng F (2013). J Anhui Agri Sci..

[4] Komar-Tyomnaya L (2015). Acta Hort. ISHS.

[5] Cao Y (2017). BMC Plant Biol..

[6] Ma R (2007). Asian Australas J Plant Sci Biotechnol..

[7] Zeng X (2016). Anhui Agri Sci Bull..

[8] Peng M (2015). Biochem System Ecol..

[9] Bao W (2017). PLoS ONE.

[10] Darwin C (1877). The Different Forms of Flowers on Plants of The Same Species.

[11] Lippman ZB, Zamir D (2007). Trends Genet..

[12] Mackenzie S (2012). Plant Biotechnology and Agriculture.

[13] Chen L, Liu YG (2014). Annu. Rev. Plant Biol..

[14] Bohra A (2016). Plant Cell. Rep..

[15] Mühleisen J (2013). Crop Sci..

[16] Yamagishi H, Bhat SR (2014). Breed Sci..

[17] Yu S (2016). Chin. Agric. Sci..

[18] Yang Y (2018). BMC Plant Biol..

[19] Timofejeva L (2013). G3 (Bethesda).

[20] https://www.intechopen.com/books/new-visions-inplant-science/plant-metabolomics-an-emergingtechnology-for-crop-improvement.

[21] Fernie AR, Schauer N (2009). Trends Genet..

[12] Resham S (2014). Emerging Technologies and Management of Crop Stress Tolerance.

[23] Ramalingam A (2015). Front Plant Sci..

[24] Kumar R (2017). Front. Plant Sci..

[25] D’Amelia L (2018). Springer Cham,.

[26] Fischer R (1997). Plant J..

[27] van Eldik GJ (1997). Plant J..

[28] Xu P (1997). Planta.

[29] Yuan H (2018). BioMed Res Int..

[30] Papini A (1999). Protoplasma.

[31] Kawanabe T (2006). Plant Cell Physiol..

[32] Sheoran S (2015). Applied Biochem Biotechnol..

[33] Shull GF (1908). Rep Am Breeders Assoc..

[34] Saxena KB, Hingane AJ (2015). Springer India.

[35] Ruiz ON, Daniell H (2005). Plant Physiology.

[36] Shukla P (2014). Funct Integr Genomics.

[37] van der Meer IM (1992). Plant Cell.

[38] Hsieh K (2007). Plant Cell..

[39] Filkowski J (2004). Plant J..

[40] Ma J (2012). PLoS ONE.

[41] Li Y (2019). Int. J. Mol. Sci..

[42] Ingrosso I (2011). Plant Physiol Biochem..

[43] Ye J (2017). Front Plant Sci..

[44] Datta R (2002). Plant Physiol..

[45] Mamun EA (2006). Cell. Biol. Int..

[46] Shishova M (2019). Metabolites.

[47] Zhao Q (2018). Plant Physiol Biochem..

[48] Qu GR (2014). J. Integr. Plant Biol..

[49] Li SQ (2004). Funct. Plant Biol..

[50] Wan CX (2007). Plant Cell Rep..

[51] Deng MH (2012). Sci. Hortic..

[52] Ba QS (2013). Can. J. Plant Sci..

[53] Ding X (2019). Int. J. Mol. Sci..

[54] Jiang PD (2007). Plant Cell Rep..

[55] Gonzalez A (2008). Plant J..

[56] Lloyd A (2017). Plant Cell Physiol..

[57] Ying H (2019). BMC Plant Biol.

[58] Zhang S (2019). Biomed Res Int..

[59] Dossa K (2019). Plant Biotechmol J..

[60] Livak KJ, Schmittgen TD (2001). Methods.

